# Efficacy of Piroxicam Mesotherapy in Treatment of Knee Osteoarthritis: A Randomized Clinical Trial

**DOI:** 10.1155/2020/6940741

**Published:** 2020-08-01

**Authors:** Hamid Reza Farpour, Fahime Estakhri, Mohadese Zakeri, Reyhaneh Parvin

**Affiliations:** ^1^Shiraz Geriatric Research Center, Department of Physical Medicine and Rehabilitation, Shiraz University of Medical Sciences, Shiraz, Iran; ^2^Bone and Joint Diseases Research Center, Shiraz University of Medical Sciences, Shiraz, Iran; ^3^Department of Physical Medicine and Rehabilitation, Shiraz University of Medical Sciences, Shiraz, Iran

## Abstract

**Introduction:**

Knee osteoarthritis (KOA) is one of the most common degenerative diseases that lead to pain and disability. Oral NSAIDs are effective drugs used to alleviate symptoms in patients with KOA, but they have several important complications, especially in the elderly. In this study, we evaluated the effectiveness of mesotherapy on pain reduction and improvement of functioning in patients with KOA.

**Methods:**

Sixty-two patients with KOA, grade 2-3 of the Kellgren–Lawrence scale, were randomized into two groups: the mesotherapy group, in which two injections were applied with piroxicam at a 10-day interval, and the oral group, in which piroxicam was prescribed for 10 days. The patients were evaluated before the treatment and 2, 4, and 8 weeks after it using the Visual Analogue Scale (VAS), Oxford Knee Scare (OKS), and Western Ontario McMaster University Osteoarthritis Index (WOMAC, Persian version).

**Results:**

There was no significant difference in demographic characteristics and baseline pain and function scores between the two groups. After 2, 4, and 8 weeks of follow-up, VAS, WOMAC, and OKS scores significantly improved in both groups (in the mesotherapy group: *p* value <0.001 in all three scores and in the oral group: *p* value <0.001 in the VAS scale and *p* value <0.05 in WOMAC and OKS scores). There was no significant difference between the two groups at any time in the VAS score, but improvement in WOMAC and OKS scales in the mesotherapy group was significantly better (*p* value <0.05 in both scales [*p* value <0.03 in OKS and *p* value <0.02 in WOMAC scales]). Side effects in both groups were not serious: limited heart burn in 32.2% of the total subjects in the oral group and pain at the injection site in 3.2% and bruises in 38.7% of the total subjects in the mesotherapy group.

**Conclusion:**

Mesotherapy is an effective and safe treatment modality in patients with mild-to-moderate KOA in the short term. This trial is registered with IRCT2017052434113N1.

## 1. Background

Knee osteoarthritis (KOA) is one of the most common chronic joint diseases. Due to aging, increase in life expectancy, and epidemic of obesity, incidence and prevalence of KOA is rising [1]. According to the survey performed in the United States, the prevalence of radiologic KOA is about 20–28%, and in symptomatic KOA, it is about 7–17% in people over 45 years [2]. Pain and loss of function are the main symptoms that lead to treatment [1]. KOA incurs significant costs economically and socially [3]. Arthritis, after headache and back lumbago, is the third common pain condition that causes loss of productive work time in the United States [4].

Pain and loss of function are the main symptoms that lead to treatment [1]. There are different nonsurgical and surgical methods for decreasing pain, improving function, and preventing or correcting deformities in KOA [5].

In conservative management, nonpharmacological options are education, exercise, weight loss, physical therapy, acupuncture, assistive devices, taping, etc. Pharmacological treatment options are topical agents (capsaicin and diclofenac sodium), systemic drugs (acetaminophen, nonsteroidal anti-inflammatory drugs [NSAIDs], opioids, and duloxetine), and localized injection therapy (peri- and intra-articular prolotherapy, PRP, hyaluronic acid, glucocorticoids, and ozone) [5–13].

One of the important challenges in the KOA management is the most effective and safe treatment for each patient [14]. Systemic NSAIDs are effective, low cost, and easy access drugs for pain relief and functional improvement in patients with KOA, but they have several side effects such as gastrointestinal and cardiovascular problems. The risk of complications on the presence of other risk factors such as aging is higher [15]. However, newer NSAIDs (for example: lornoxicam) have better effects on pain and function with lesser complications in patients with KOA [16].

Mesotherapy is a procedure used to inject pharmacological substances or plant extracts into the dermis or subcutaneous layer of the skin. This technique was invented by Pistor in 1958 for the treatment of musculoskeletal pain and vascular and infectious diseases [17–19]. “One of the main advantages of mesotherapy is that local pharmacological effects can be obtained without the need for high systemic concentration. Mesotherapy in combination with systemic therapy can produce synergistic effects” [19, 20]. Side effects relative to systemic therapy can be reduced due to local injection of drugs. Adverse effects of mesotherapy depend on the technique and active substance, such as allergic reactions, pain, ecchymosis, bleeding, and infection. These side effects are often transitory and reversible [18, 19].

Mesotherapy is a type of alternative therapy used to control the local pain [21]. In one study, this method reduced cervical and low back pain by at least 50% compared to the baseline [19]. In another study, mesotherapy with NSAIDs and corticosteroids in acute low back pain could provide the same therapeutic effects as that induced by systemic drug administration [22]. In one study, about pes anserine bursitis in symptomatic osteoarthritis patients, mesotherapy with diclofenac reduced pain and ultrasonographic findings significantly [20]. In a study on patients with KOA, mesotherapy with different protocols depending on the acute or chronic phase of OA in six special points around the knee was done and compared with oral administration of diclofenac for three months. Both treatments improved the patients' clinical conditions, but mesotherapy was more efficient in WOMAC scores with fewer side effects [17].

There are few surveys and specific protocols for treatment of KOA symptoms with mesotherapy. Thus, we aimed to compare the efficacy of mesotherapy with piroxicam and short-term prescription of this drug on decreasing pain and improving the functions of patients with KOA.

## 2. Materials and Methods

### 2.1. Trial Design

**Figure 1:**
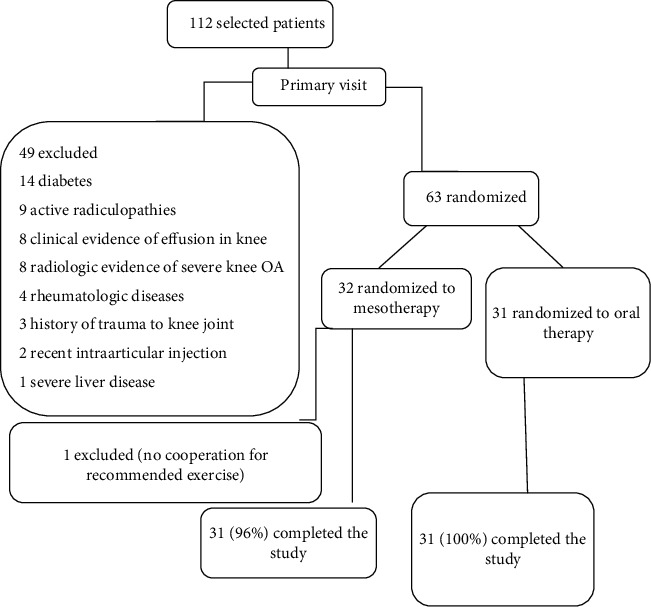
Study consort flowchart.

This study was a single-blinded, randomized clinical trial with a blocked randomization and parallel design. The consort flowchart is illustrated in Figure 1.

### 2.2. Participants

Participants in this study were selected from patients with knee pain that referred to physical medicine and rehabilitation clinics in Shahid Rajaee, Shahid Faghihi, Imam Reza, and Hafez hospitals (academic centers of the Shiraz University of Medical Sciences [SUMS]) between September 2017 and January 2018.

### 2.3. Selection Criteria

The inclusion criteria were clinical symptoms of KOA (based on the clinical criteria of the American College of Rheumatology [1]) with a symptom duration of more than 3 months, grade 2 or 3 (mild or moderate) of the Kellgren–Lawrence scale in X-ray [23] within the past 1 month, age of 40–70 years in both sexes, Visual Analogue Scale of more than three [24], absence of any pathologic conditions around the knee such as bursitis or cellulitis, and willingness to participate in the study. Exclusion criteria included any intra- or periarticular injection during the past three months; use of NSAIDs for pain relief during the past three days; a history of cancer, diabetes mellitus, rheumatologic and collagen-vascular disease, and gout; clinical signs of effusion in the knee, hotness or redness at the knee, history of trauma to the knee; history of knee arthroplasty or other surgeries; BMI > 42; presence of active radiculopathy, myopathy, or peripheral neuropathy in lower limbs; pregnancy or breastfeeding; allergy to piroxicam and other NSAIDs; severe gastrointestinal, hepatic, renal, respiratory, cerebral, and cardiovascular diseases; severe KOA in X-ray (grade 4 of the Kellgren–Lawrence scale); any condition with bleeding tendency; administration of anticoagulant drugs; any serious systemic or local infection (such as brucellosis); inability to complete the questionnaires.

### 2.4. Intervention

#### 2.4.1. Mesotherapy Group

Patients received subcutaneous injection by using the disposable sterile syringes (thin needle: 0.27 mm × 4 mm) containing a mix of 20 mg/1 ml piroxicam (Roxicam^R^, manufactured by OSVAH Pharma. Co., Tehran, Iran) and 2 ml of 2% lidocaine (Xylex^R^, manufactured by Sinadarou Pharma. Co., Tehran, Iran) in painful points around the knee (2–6 points). The needle angle when entering the skin was 30–45 degrees. Before the injections, the skin was prepped and draped. After 10 days of injection, it was repeated (each patient received two sessions of mesotherapy). The patients were trained for lifestyle modification and quadriceps femoris strengthening exercise (with contraction of the muscle) and hamstring and calve muscles stretching (with straight leg rising to 60–70 degrees) [25]. Duration of each exercise was 15–30 seconds and repeated 10–30 times a day according to the patient tolerance. Patients were advised to avoid any other treatment for KOA.

#### 2.4.2. Oral Administration Group

Patients received an oral administration of 2 capsules of piroxicam (piroxicam 10 mg, manufactured by Zahravi Pharma. Co., Tabriz, Iran) once every day for 10 days. All patients were trained for lifestyle modification and the same exercises as the mesotherapy group. Patients were advised to avoid any other treatment for KOA.

All patients in both groups were given a phone number to ask any probable question. The treatment complications were considered in each follow-up.

### 2.5. Outcomes

All patients were evaluated before treatment and 2, 4, and 8 weeks after treatment by the Visual Analogue Scale (VAS), Western Ontario and McMaster Universities Arthritis Index (WOMAC), and Oxford Knee Scale (OKS) questionnaires. VAS evaluates the pain intensity with 10 degrees from 0 (no pain) to 10 (most possible pain) [24]. WOMAC is an index for evaluation of function with three parts: pain with five items, joint stiffness with 2 items, and physical function with 17 items (total WOMAC score was 24 items). Each item includes 5 scales from 0 to 4 (none: 0, mild: 1, moderate: 2, severe: 3, and extreme: 4). The total WOMAC score is defined between 0 as the best function and 96 as the worst function [26]. Also, OKS evaluates the function of the patient and contains 12 items with 5 scores from 0 to 4 (none: 4, very mild: 3, mild: 2, moderate: 1, and severe: 0); score 0 points to the worst function and score 48 refers to the best performance [27]. According to previous studies, there is no limitation to the use of questionnaires for one person's knees [26, 27]. The most important demographic criteria were age, sex, and body mass index (BMI).

### 2.6. Sample Size

The sample size was determined based on the statistical analysis considering data from similar studies with a significance level of 0.5(*p* < 0.05), confidence interval of 95%, power of 1.20, and probable dropout rate of 20%. The number of samples in each group was 30 (each knee was considered as a sample).

### 2.7. Randomization and Blinding

63 patients were randomly divided into two parallel groups (group A: mesotherapy and group B: oral therapy). We used restricted block randomization. The statistician was unaware of allocation (single-blind study).

### 2.8. Statistical Method

All data were analyzed using SPSS (Statistical Package for the Social Science) software version. 22. Normality of the data was evaluated by Shapiro–Wilk's test. To evaluate the changes in data over the time, the Freidman test and the chi-square (*X*^2^) index were applied. To compare the two groups, we used the independent *t*-test. Standard deviation, mean, and confidence interval were calculated, and finally statistical significance was considered as a *p* value <0.05. The Wilcoxon signal ranks test and Bonferroni correction were used for comparison of 6 time points (time 1: before treatment and 2^nd^ week after treatment, time 2: before treatment and 4^th^ week after treatment, time 3: before treatment and 8^th^ week after treatment, time 4: 2^nd^ and 4^th^ weeks after treatment, time 5: 2^nd^ and 8^th^ weeks after treatment, and finally time 6: 4^th^ and 8^th^ weeks after treatment) in each group, and the Bonferroni-corrected *p* value was considered as <0.008(0.05 ÷ 6).

### 2.9. Ethical Considerations

We explained both methods and side effects to each patient before participating in the study. All patients gave their informed consent form. They could withdraw from the study whenever they wanted. All patients were trained on lifestyle modification and appropriate exercise for KOA (standard and basic treatments). All complications were carefully followed.

This study was approved by the Medical Ethics Committee of the Shiraz University of Medical Sciences (SUMS) with the ethics number: IR.SUMS.MED.REC.1395.59 and registered with a registration ID of 2017052434113N1 at the IRCT (Iranian Registry of Clinical Trials).

## 3. Results

We assessed 112 patients with knee pain who were the candidates to participate in our study. Forty-nine patients had at least one exclusion criterion and were not included. Other patients (*n* = 63) were randomized to two groups: mesotherapy (group A) and oral therapy (group B). One patient from the mesotherapy group withdrew from the study. Finally, 31 patients in the mesotherapy group and 31 in the oral therapy group completed the study. There was no statistically significant difference in age, sex, BMI (Table 1), VAS, OKS, and WOMAC scores before the treatment (Table 2).

The VAS score was decreased in both groups after 2, 4, and 8 weeks of follow-up (*p* value <0.001). There was no significant difference between the two groups at any time (*p* value >0.05) (Table 3), but pain decrement compared to the baseline was more in the mesotherapy group in the fourth week (*p* value <0.001) (Table 4). Data analysis showed that the OKS score improved in both groups after 2, 4, and 8 weeks of follow-up (*p* value <0.05), but this increment was more in the mesotherapy group (*p* value<0.05) (Table 3). For both the VAS score and the OKS score, at all times except time 6 in the mesotherapy group and time 4 in the oral group, the Bonferroni-corrected *p* value was <0.008. These results were also applied to the change in function compared to the baseline (Table 4). The results of the WOMAC analysis showed that pain and function and total score improved significantly in both groups after 2, 4, and 8 weeks of follow-up (*p* value <0.05) with better results in the mesotherapy group (*p* value <0.05), but improvement in the joint stiffness occurred only in the mesotherapy group (*p* value <0.001) (Table 5). Although changes in pain, function, and the total WOMAC score, compared to the baseline, were clearly observed in both groups (*p* value <0.05), the changes in the mesotherapy group were more significantly different (*p* value <0.001) (Table 6). For the total WOMAC score, at all times except time 6 in the mesotherapy group, the Bonferroni-corrected *p* value was <0.008.

## 4. Discussion

There are important limitations for systemic therapy in patients with KOA, especially in the elderly. In this study, we evaluated the effectiveness of mesotherapy with piroxicam (as a local treatment) on pain reduction and functional improvement in patients with KOA and compared it with oral piroxicam prescription. The study's results showed that mesotherapy with piroxicam can reduce the pain similar to administration of oral piroxicam at 8 weeks of follow-up. Both methods improved the function (OKS and WOMAC scales) in this time, but effects were better in the mesotherapy group (perhaps due to higher drug concentration at the local area in the mesotherapy procedure). There was no significant difference in VAS, OKS, and WOMAC scores between the fourth and eighth weeks after treatment in the mesotherapy group. In the oral group, this was true for VAS and OKS scores between the second and fourth weeks after treatment. In our study, there were no serious side effects. In the oral therapy group, 8 patients (10 knees, 32.2% of the total subjects) had heart burn which disappeared with 3 doses of ranitidine for 7 days. Pain at the injection site is one of the most common complications of mesotherapy. In our study, one patient (3.2% of the total subjects) reported pain for one day at the injection site that improved with ice massage and 2 doses of 500 mg acetaminophen. Bruises were created at the injection site on 12 knees (38.7% of the total subjects) that resolved to a maximum of 4 weeks. Complications such as bleeding or hypersensitivity reactions were not observed.

In another study on the efficacy and safety of mesotherapy in patients with KOA (published in 2018) conducted by Chen et al., it was shown that oral therapy with diclofenac (75 mg twice per day for 3 months) and mesotherapy with 2 protocols for acute (40 mg piroxicam, 100 units of calcitonin, and 2 cc lidocaine 1%) and chronic conditions (2 cc procaine 2%, 2 cc organic silica, and 100 units of calcitonin) significantly improved biochemical markers and clinical conditions. Mesotherapy had significantly fewer side effects and was more efficient in terms of hematology and WOMAC scores [17]. Saggini et al. conducted a study in 2015 to compare the efficacy of mesotherapy with 25 mg diclofenac, nine sessions, and oral diclofenac (50 mg once a day for 3 weeks) on pes anserine bursitis in KOA (grade 2 of Kellgren–Lawrence classification). In all 112 patients, pain level decreased significantly after treatment. Ultrasonography showed a reduction in the hypoechoic area related to bursitis only in the mesotherapy group [20].

There are studies that support the good effect of mesotherapy in reducing back pain [19, 22].

In our study, sessions of mesotherapy were limited (only 2 sessions) and oral piroxicam was prescribed only for 10 days (20 mg once a day). This study showed that mesotherapy was an effective and safe procedure in patients with mild or moderate KOA.

The limitations of our study included the lack of objective evaluation of the effects of interventions, short-term follow-up, lack of measurement of drug plasma level, and lack of a placebo group.

## 5. Conclusion

Mesotherapy is an effective and well-tolerated method for pain reduction and function improvement in patients with mild-to-moderate KOA in the short term and can be considered as an alternative therapy in patients with contraindications for systemic therapy with NSAIDs. These results are based on subjective findings. Further studies are required to be conducted to evaluate mesotherapy with different methods, long-term follow-up, and objective findings.

## Figures and Tables

**Table 1:** Baseline characteristics of patients.

	Mesotherapy group	Oral therapy group	*p* value
Sex	F^a^: 29 (93.5%) M^b^: 2 (6.5%)	F: 25 (80.6%) M: 6 (19.4%)	0.09
Age, mean ± SD^c^	56.87 ± 9.14	53.45 ± 8.63	0.66
BMI^d^ (kg/m^2^), mean ± SD	27.61 ± 5.08	28.3 ± 4.66	0.15

^a^F, female; ^b^M, male; ^c^SD, standard deviation; ^d^BMI, body mass index.

**Table 2:** Baseline VAS, OKS, and WOMAC scores.

	Mesotherapy group	Oral therapy group	*p* value
VAS^a^, mean ± SD^b^	8.25 ± 1.99	7.45 ± 1.68	0.07
OKS^c^, mean ± SD	22.93 ± 7.57	21.13 ± 4.54	0.11
WOMAC^d^, mean ± SD			
Pain	16.0 ± 3.49	14.96 ± 3.33	0.13
Stiffness	4.03 ± 1.94	3.97 ± 1.34	0.66
Function	33.35 ± 9.85	31.51 ± 9.75	0.12
Total score	53.38 ± 14.41	50.44 ± 14.14	0.17

^a^VAS, Visual Analogue Scale; ^b^SD, standard deviation; ^c^OKS, Oxford Knee Scale; ^d^WOMAC, Western Ontario and McMaster Universities Arthritis Index.

**Table 3:** VAS and OKS scores in both groups during the follow-up.

	Mesotherapy group	Oral therapy group	*p* value (within groups)	*p* value (between groups)
VAS^a^, mean, SD^b^			0.001	
Baseline	8.25 ± 1.99	7.45 ± 1.68		0.07
2^nd^ week	5.25 ± 1.99	5.22 ± 2.27		0.95
4^th^ week	3.51 ± 2.17	4.48 ± 2.06		0.07
8^th^ week	3.12 ± 2.09	3.38 ± 2.02		0.62

OKS^c^, mean ± SD			Mesotherapy group: 0.001 Oral therapy group: 0.04	
Baseline	22.93 ± 7.57	21.13 ± 4.54		0.11
2^nd^ week	31.60 ± 8.28	25.33 ± 5.93		0.03
4^th^ week	38.41 ± 9.12	27.25 ± 6.99		0.01
8^th^ week	39.05 ± 15.32	30.22 ± 8.10		0.01

^a^VAS, Visual Analogue Scale; ^b^SD, standard deviation; ^c^OKS, Oxford Knee Scale.

**Table 4:** VAS score decrement and OKS score increment in both groups during the follow-up compared to the baseline.

	Mesotherapy group	Oral therapy group	*p* value (within groups)	*p* value (between groups)
VAS^a^ decrement, mean ± SD^b^			0.001	
In 2 weeks	3.00 ± 1.86	2.22 ± 2.24		0.14
In 4 weeks	4.74 ± 2.26	2.96 ± 1.45		0.001
In 8 weeks	5.12 ± 2.49	4.06 ± 2.76		0.11

OKS^c^ increment, mean ± SD			Mesotherapy group: 0.001 Oral therapy group: 0.03	
In 2 weeks	8.67 ± 5.00	4.19 ± 5.49		0.001
In 4 weeks	15.48 ± 7.11	6.12 ± 5.53		0.001
In 8 weeks	16.12 ± 9.39	9.09 ± 7.02		0.001

^a^VAS, Visual Analogue Scale; ^b^SD, standard deviation; ^c^OKS, Oxford Knee Scale.

**Table 5:** WOMAC score in both groups during the follow-up.

	Mesotherapy group	Oral therapy group	*p* value (within groups)	*p* value (between groups)
WOMAC^a^, mean ± SD^b^				
Pain			Mesotherapy group: 0.001 Oral therapy group: 0.04	
Baseline	16.00 ± 3.49	14.96 ± 3.33		0.13
2^nd^ week	11.13 ± 2.89	13.26 ± 3.01		0.04
4^th^ week	8.04 ± 2.40	12.64 ± 2.98		0.01
8^th^ week	7.55 ± 2.40	11.38 ± 2.90		0.01
Stiffness			Mesotherapy group: 0.001 Oral therapy group: 0.06	
Baseline	4.03 ± 1.94	3.97 ± 1.34		0.66
2^nd^ week	3.07 ± 1.23	3.78 ± 1.25		0.03
4^th^ week	2.36 ± 0.61	3.68 ± 1.15		0.01
8^th^ week	2.33 ± 0.65	3.36 ± 1.05		0.02
Function			Mesotherapy group: 0.001 Oral therapy group: 0.03	
Baseline	33.35 ± 9.85	31.51 ± 9.75		0.12
2^nd^ week	18.32 ± 8.19	24.68 ± 8.11		0.04
4^th^ week	9.45 ± 6.48	16.45 ± 8.09		0.03
8^th^ week	8.74 ± 7.07	12.63 ± 7.78		0.04
Total score			Mesotherapy group: 0.001 Oral therapy group: 0.04	
Baseline	53.38 ± 14.41	50.44 ± 14.14		0.17
2^nd^ week	32.52 ± 10.11	41.72 ± 10.76		0.02
4^th^ week	19.85 ± 6.78	32.77 ± 10.34		0.001
8^th^ week	18.62 ± 6.11	27.37 ± 9.55		0.02

^a^WOMAC, Western Ontario and McMaster Universities Arthritis Index; ^b^SD, standard deviation.

**Table 6:** WOMAC score improvement in both groups during the follow-up compared to the baseline.

	Mesotherapy group	Oral therapy group	*p* value (within groups)	*p* value (between groups)
WOMAC^a^ improvement, mean ± SD^b^				
Pain decrement			Mesotherapy group: 0.001 Oral therapy group: 0.04	
In 2 weeks	4.87 ± 3.05	1.70 ± 2.84		0.001
In 4 weeks	7.96 ± 3.19	2.32 ± 2.84		0.001
In 8 weeks	8.45 ± 4.28	3.58 ± 3.37		0.001
Stiffness decrement			Mesotherapy group: 0.001 Oral therapy group: 0.06	
In 2 weeks	0.96 ± 1.16	0.19 ± 0.54		0.001
In 4 weeks	1.67 ± 1.72	0.29 ± 0.58		0.001
In 8 weeks	1.70 ± 1.69	0.61 ± 1.17		0.004
Function improvement			Mesotherapy group: 0.001 Oral therapy group: 0.03	
In 2 weeks	15.03 ± 8.59	6.83 ± 9.82		0.001
In 4 weeks	23.90 ± 10.03	15.06 ± 8.60		0.001
In 8 weeks	24.61 ± 12.25	18.88 ± 9.52		0.001
Total score improvement			Mesotherapy group: 0.001 Oral therapy group: 0.04	
In 2 weeks	20.86 ± 11.93	8.72 ± 4.36		0.001
In 4 weeks	33.53 ± 13.96	17.67 ± 7.11		0.001
In 8 weeks	34.76 ± 17.14	23.07 ± 10.52		0.001

^a^WOMAC, Western Ontario and McMaster Universities Arthritis Index; ^b^SD, standard deviation.

## Data Availability

The data used to support the findings of this study are available from the corresponding author upon request.
